# Low salt exposure results in inactivation of *Toxoplasma gondii* bradyzoites during formulation of dry cured ready-to-eat pork sausage

**DOI:** 10.1016/j.fawpar.2019.e00047

**Published:** 2019-03-14

**Authors:** J. Fredericks, D.S. Hawkins-Cooper, D.E. Hill, J. Luchansky, A. Porto-Fett, H.R. Gamble, V.M. Fournet, J.F. Urban, R. Holley, J.P. Dubey

**Affiliations:** a USDA, ARS, NEA, Animal Parasitic Diseases Laboratory, BARC-East, Bldgs. 1001 & 307-C, Beltsville, MD 20705, United States of America; b USDA, ARS, NEA, Food Safety and Intervention Technologies, 600 E. Mermaid Ln. ERRC, Wyndmoor, PA 19038-8598, United States of America; c National Academy of Sciences, 500 Fifth Street NW, Washington, DC 20001, United States of America; d USDA, ARS, BHNRC, Diet, Genomics, and Immunology Laboratory, BARC-East, Bldgs. 307-C, Beltsville, MD 20705, United States of America; e Faculty of Agricultural and Food Sciences, Room 250 Ellis Building, 13 Freedman Crescent, University of Manitoba, Winnipeg, MB R3T 2N2, Canada

**Keywords:** *Toxoplasma gondii*, Dry cured sausage, Pepperoni, Ready to eat meat

## Abstract

The production of safe and healthy food products represents one of the main objectives of the food industry. The presence of microorganisms in meat and products containing meat can result in a range of human health problems, as well as economic losses to producers of these products. However, contaminated meat products continue to initiate serious and large-scale outbreaks of disease in consumers. In addition to outbreaks of diseases caused by bacteria and viruses, parasitic organisms, such as *Toxoplasma gondii*, are responsible for foodborne infections worldwide, and in the case of *T. gondii*, is considered the 2nd leading cause of death from foodborne illness in the U.S. Transmission of *Toxoplasma gondii* has historically been linked to the consumption of raw or undercooked meat products, including pork. Specific concerns with respect to pork products are ready-to-eat (RTE) pork meals. These are pork or products containing pork that are prepared by curing or drying, and are not intended to be cooked before being consumed.

Previous studies have demonstrated that *T. gondii* is inactivated during dry cured sausage preparation, apparently in the batter during fermentation. In this study, we have analyzed timing of inactivation of *T. gondii* in freshly prepared pepperoni batter to confirm our previous findings, to determine how quickly inactivation occurs during fermentation, and to confirm what parameters of the sausage preparation are involved in inactivation of the parasite. Results from the current and previous study indicate that rapid inactivation of *T. gondii* bradyzoites occurs in low salt batter for dry cured sausage within 4 h of initiation of fermentation.

## Highlights

• Pork containing *Toxoplasma* bradyzoites was used to prepare dry cured pepperoni sausage.
• A low salt (1.3%) formulation was used to prepare the pepperoni batter.
• *T. gondii* was inactivated in pepperoni batter in <4 h of fermentation.
• Risk to consumers of ready to eat (RTE) dry cured pepperoni from *T. gondii* is low.
• The safety of RTE products using industry standard dry cure procedures was confirmed.

## 1 Introduction

Toxoplasmosis is a zoonotic disease that occurs worldwide and is caused by the protozoan parasite *Toxoplasma gondii*. *Toxoplasma* has three infectious stages: the rapidly dividing tachyzoite, the slow growing bradyzoite (in tissue cysts), and the quiescent sporozoite (in oocysts) (Dubey et al., 1998). *Toxoplasma* has several potential routes of transmission between different host species. For example, *T. gondii* may be horizontally transmitted, which may involve any of the three life-cycle stages, i.e. ingesting infectious oocysts from the environment, ingesting tissue cysts containing bradyzoites in meat, or ingesting tachyzoites circulating in meat (Tenter et al., 2000; Dubey, 2010). Though oocyst transmission is thought to contribute significantly to human infection (Munoz-Zanzi et al., 2010; Hill et al., 2011; Boyer et al., 2011), the consumption of raw or undercooked contaminated pork meat is a source of infection that has been regarded as a major route of *T. gondii* transmission to humans (Gamble, 1997; Scallan et al., 2011).

Ready-to-eat meats are a special concern since these may be consumed without further cooking and have been known to act as a vehicle for transmission of pathogenic microorganisms to consumers (Syne et al., 2013; Cardoso-Toset et al., 2017; Morales-Partera et al., 2017). Given the possibility of the presence of *T. gondii* in pork meat, the frequent use of pork for RTE products increases the potential risk for transmission of these parasites to consumers.

Current meat-curing practices have beneficial effects on meat products, such as inhibiting the growth of microorganisms, and increasing shelf life and stability (Getty, 2005). Curing processes for pork meat in the U.S. once required individual validation of methods to demonstrate inactivation of *Trichinella spiralis* (9 CFR 318.10); these regulatory requirements have recently been rescinded in favor of an approach driven by HACCP (hazard analysis and critical control point) considerations for *T. spiralis* as a risk reasonably likely to occur. It was assumed, with little evidence, that the curing processes required to inactivate *T. spiralis* also inactivated *T. gondii*. However, for protozoan parasites such as *T. gondii*, no strictures or regulatory framework exists, and no model of meat chemistry that can be correlated with inactivation of *T. gondii* is in place. Given the possibility of the presence of *T. gondii* in pork meat, and the frequent use of pork for RTE products not intended to be cooked, curing methods which inactivate *T. gondii* early in the curing process would be of great value to producers. A previous study (Hill et al., 2018) demonstrated that *T. gondii* is inactivated during dry cured sausage preparation. *Toxoplasma gondii* was completely inactivated in sausage batter during the fermentation process. The study determined that of the 5 variables evaluated (time, temperature, water activity, pH, and NaCl concentration), NaCl concentration was the determining factor in speed of inactivation, even in a relatively low salt batter containing 1.3% NaCl. In this study, we evaluated the timing of inactivation of *T. gondii* bradyzoites in sausage batter during fermentation using the low salt concentration identified in the previous study (1.3% NaCl) during preparation of dry cured pork sausage, presuming that this low salt concentration would provide the best-case scenario for survival of bradyzoites in sausage batter during a dry cure process.

## 2 Materials and methods

For experimental data collection, a total of 10 mixed breed pigs (Pig Improvement Company (PIC, Hendersonville, TN), proprietary breeding background), 10–12 weeks of age each, were inoculated subcutaneously with 10,000 VEG strain *T. gondii* bradyzoites freed from the tissue cyst. Tissue cysts were isolated from the brains of *T. gondii* infected mice inoculated (per os) 45 days previously with 50 VEG strain oocysts (BAACUC Approval # 16-014 (mice). Infected pigs were housed at the USDA's Beltsville Agricultural Research Center, and grown to near market size (67 to 75 kg). Infected pigs were housed in separate pens and quarantined from non-infected pigs in accordance with the Animal Welfare Act, Guide for the Care and Use of Laboratory Animals (https://www.nap.edu/search/?term=Guide+for+the+Care+and+Use+of+Laboratory+Animals+) and with the approval of the USDA/ARS Beltsville Area Animal Care and Use Committee (BAACUC, Approval # 15-014 (pigs)). After 60 days, infected pigs were humanely euthanized by electrocution followed by exsanguination. Whole blood was collected into 50 ml tubes and allowed to coagulate at room temperature. Serum was isolated from whole blood by centrifugation at 293 ×*g*. Serum was collected and stored frozen until used. Serum was tested in duplicate for antibody to *T. gondii* by the modified agglutination test (MAT; Dubey, 2010). Titers over 1:25 were considered positive.

The triceps, picnics, hams, neck, rump, and loins were collected from all pigs and hand trimmed of excess fat and connective tissue to yield a total of 23 to 30 kg of meat from each animal. Back fat was also collected from each animal and supplemented with purchased pork back fat (C & C Meats, Upper Marlboro, MD) for sausage preparation. A flow chart for preparation of dry cured sausage is available (https://www.fsis.usda.gov/shared/PDF/FSRE_SS_6DriedMeatsProcessing.pdf?redirecthttp=true). The starting pH of the collected meat was pH 6.91.

The blend of pork meat and chilled fat was coarse ground using a sanitized mixer/grinder through an accessory grinding plate with 9.5 mm holes (Model 4346; Hobart Corporation, Troy, OH, USA), and mixed for 20 min at 4 °C to provide a uniform distribution of parasites. This was the initial ground pork meat preparation.

The mixer/grinder was sanitized with a quaternary ammonium-based germicide, multipurpose detergent (Misty® Biodet ND32), rinsed thoroughly for 3 min with hot water (87.8 °C), and then allowed to air dry at room temperature for up to 1 h.

Pepperoni batter was prepared using *T. gondii* infected ground pork muscles described above and pork back fat to achieve a ratio of about 70% lean meat to 30% fat. The coarse ground pork was re-loaded into the sanitized mixer/grinder, reground to produce to ~3 mm particles, and dry ingredients were added to achieve combinations of salt (1.3%; high-grade sodium chloride, The Canadian Salt Company Ltd., Pointe Claire, QC, Canada), dextrose/sugar at 0.7% (for pH endpoint 4.6) or 0.25% (for pH endpoint 5.2) to achieve common curing method endpoints for pH (Tate & Lyle of Nealanders International Inc., Mississauga, ON, Canada), 0.05% sodium erythorbate, and 0.25% cure (sodium erythorbate and sodium nitrate/nitrite; Wiberg Canada Corporation, Oakville, ON, Canada). The pepperoni batter was mixed for about 1 min before the addition of starter culture mixture (*Pediococcus acidilactici* and *Staphylococcus carnosus*) in water (Formula 102; Trumark Inc., Linden, NJ, USA), per the manufacturer's instruction to yield about 6 to 7 log10 CFU/g, respectively. Approximately 100 kg of raw pepperoni batter was prepared in each of 2 batches (0.7 or 0.25% dextrose; containing *T. gondii* infected pork) as described below, enough for 200 pepperoni chubs weighing 500 g each from each of the 2 batter preparations.

The presence of viable *T. gondii* tissue cysts in the initial ground pork meat and batter mixtures (before beginning of fermentation) was determined by digestion of a 100 g sample of the initial ground pork meat preparation, and a 100 g sample of each of the 2 batter mixtures in acidified pepsin (porcine stomach pepsin 1:10,000 granular, American Laboratories Incorporated Omaha, Nebraska) for isolation of bradyzoites (Hill et al., 2018). The digest sediment was neutralized with 15 ml of freshly prepared 1.2% sodium bicarbonate (pH 8.3) with phenol red. After mixing, samples were centrifuged at 2000 ×*g* for 10 min. The final sediment was diluted in 10 ml of sterile saline containing 1000 units of penicillin and 100 μg of streptomycin per ml. 1 ml of the diluted sediment was subcutaneously inoculated into each of ten 6–8-week-old female Swiss-Webster mice. Mice were maintained for 45 days, then euthanized. Brain squashes were prepared from each mouse; these were microscopically examined for tissue cysts to determine the presence of viable *T. gondii* in the initial meat and the freshly prepared batters used to prepare pepperoni chubs. Mice were also tested serologically for antibodies to *T. gondii* using a commercial ELISA kit (IVD Research, Carlsbad, CA), and serum collected during necropsy. The manufacturer's instructions were essentially followed with the addition of mouse specific reagents (use of Horseradish peroxidase conjugated goat anti-mouse IgG (H + L) as the second step antibody; SeraCare/KPL, Gaithersburg, MD); optical densities in excess of 0.30 were considered positive for anti-*T. gondii* antibodies.

After removal of the initial 100 g samples described above, the pepperoni batter was blended for an additional 10 min to assure uniform distribution of additives, fine ground through a 4.7 mm accessory grinding plate, then extruded into water-softened 55 mm diameter cellulose-based casings (Naturin R2; Weinheim, Germany) using either a stainless steel table- top manual piston stuffer (9 kgs capacity; Model FD-9051200; F. Dick, Esslingen, Germany) or a floor model, hydraulic-driven piston stuffer (18.6 kgs capacity; Model SC-50, Koch Equipment, Kansas City, MO, USA), to form pepperoni chubs 254 mm in length, and weighing 500 g each. After stuffing, the pepperoni chubs were hand tied with twine, transferred to a temperature- and humidity-controlled walk-in incubator (EJS Systems Inc., Chagrin Falls, OH, USA) with an air flow of 1.0 to 1.5 m/s, and hung vertically on racks so that the individual chubs were not touching. The relative humidity (RH; 88%) and air temperature (23.8oC) were controlled and constantly monitored during fermentation using the Dynamist 2000 System and Partlow MRC5000 chart recorder (EJS Systems Inc., Chagrin Falls, OH, USA). The pepperoni chubs were fermented using constant temperature, degree·hour (the time in hours at a particular temperature multiplied by the degrees in excess of 15.6oC (the critical temperature at which *Staphylococcal* growth effectively begins), and relative humidity to achieve target pH endpoints of 5.2 and 4.6. At 2 hr intervals, 100 g were collected from each of 3 chubs representing the same batter formulation and parasite infection (endpoint pH 4.6 and pH 5.2; *T. gondii* infected batter; total of 6 chubs collected at each 2 hr interval) and digested as above. 1 ml of the final digest sediments were inoculated into each of 10 mice as described above to determine continued infectivity of *T. gondii* bradyzoites exposed to conditions of batter formulation and the fermentation processes. Samples were processed as above through the desired endpoint pH (either pH 4.6 or pH 5.2), for 24 h. To determine progress of fermentation, pH, and water activity (αw) at each sampling time, 50 g samples from each of the 6 chubs described above were homogenized in 100 ml of deionized water for 1 min in a stomacher blender (Stomacher 3500, Thomas Scientific, Swedesboro, NJ, USA), and the pH of each sample was measured by immersing the electrode of the pH meter in the sample homogenate (Mettler Toledo pH/ion meter, Model 235, Schwerzenbach, Switzerland). Water activity was also recorded at the time of sampling. The αw of the chubs was measured by placing 20 g samples from individual chubs from each treatment into a sampling cup and analyzing using an electronic water activity meter (Model HP23AW, Rototronic, Hauppauge, NY, USA) calibrated to 80% RH.

Digest inoculated mice were necropsied at 45 days (putative *T. gondii* infected) and brains were examined as described above for the presence of tissue cysts. Anti-*Toxoplasma* ELISA was performed on each mouse serum to detect antibody to *T. gondii* as described above.

A binomial distribution was calculated using data generated in the mouse bioassay for detection of inactivation of *T. gondii* to determine the probability of finding no *T. gondii* infected animals in inoculated, examined mice.

## 3 Results

Analysis of MAT results from serum collected from *T. gondii* infected pigs at necropsy revealed anti-*Toxoplasma* titers ranging from 1:800 to 1:3200 in 9 of the 10 infected pigs. Meat from only these 9 pigs were used to prepare the batter. When digests from these pigs were inoculated into mice, *T. gondii* tissue cysts were observed in brain smears from 9 of 10 mice inoculated with the initial ground meat preparation; in addition, ELISA results from these 9 mice were positive. Pepperoni batters were stuffed into 55 mm casings and fermented beginning at Time-0 (T-0), continuing for 24 h, and sampled at 2 hr intervals. Four of 10 mice inoculated with digests of T-0 of fermentation, pH 5.2 endpoint batter were *T. gondii* positive both by the presence of observed tissue cysts in brain smears and ELISA serology. All mice inoculated with digests of T-0 of fermentation, pH 4.6 endpoint batter were *T. gondii* negative both by brain cyst presence and by ELISA serology. Tissue cysts were found in the brains of all positive mice, and serology results (all positive OD values were >0.428, all negative values were <0.204; established cutoff was 0.300) were concordant with brain cyst detection. All subsequent inoculations of mice resulted in negative brain smears and ELISA at all sampled time points through the 24 hour fermentation period (Table 1).

**Table 1:** Starting meat and pre-fermentation batter meat chemistry, and observed *T. gondii* tissue cysts, and ELISA results in inoculated mice, 45 days PI. Nine of 10 mice inoculated with digests of the starting meat preparation had observed tissue cysts and were serologically positive with no discordant results. Four of 10 mice inoculated with digests of T-0 fermentation, pH 5.2 endpoint batter were *T. gondii* positive both by the presence of tissue cysts and serologically with no discordant results. All 10 mice inoculated with digests of T-0 fermentation, pH 4.6 endpoint batter were *T. gondii* negative both serologically and by the presence of tissue cysts with no discordant results. All other inoculated mice were negative at all sampled time points through the 24 hour fermentation period.

Starting meat^a^				Mice inoculated, n = 10				
	% NaCl	α_w_	pH	Brain cyst/ELISA OD	Brain cyst/ELISA OD	Brain cyst/ELISA OD	Brain cyst/ELISA OD	Brain cyst/ELISA OD
	N/A	0.99	6.94	pos/1.385	pos/1.369	pos/0.654	pos/0.428	pos/0.731
				pos/1.309	pos/1.462	pos/0.578	pos/0.798	neg/0.203

Batter formulation, sampled at T-0								
				Mice inoculated, n = 10				
pH endpoint 4.6	1.3%	0.96	6.91	neg/0.203	neg/0.114	neg/0.154	neg/0.178	neg/0.128
				neg/0.125	neg/0.131	neg/0.162	neg/0.158	neg/0.142
				Mice inoculated, n = 10				
pH endpoint 5.2	1.3%	0.98	6.91	pos/0.604	neg/0.157	pos/0.571	neg/0.165	neg/0.178
				neg/0.162	pos/0.801	pos/0.539	neg/0.175	neg/0.138

a After harvesting and grinding, no additives.

The number of mice positive for *T. gondii* tissue cysts was reduced 100% (pH 4.6) and 44% (pH 5.2) in mice inoculated with digests from T-0 chubs when compared to results from mice inoculated with digests from the initial meat preparations (*p <* 0.05), and was reduced 100% after 2 h of fermentation in both batter formulations, regardless of the pH or the αw of the chubs. Based on the binomial distribution, there was a 95% chance of finding 0 out of 20 mice were infected (2 hour results only; true infection rate <0.00256) and 0 out of 240 mice were infected (averaged over all hours (2–24 h); true infection rate <0.00021). Due to sampling logistics, there were no results for times between 0 and 2 h that could have been used to estimate the decrease in positive mice during this time period. Thus, the 0.00256 true infection rate was an upper limit on the proportion of positive mice at 2 h, and this upper limit on the true infection rate decreased with additional time until it reached 0.00021 for 24 h.

Water activity in all chubs remained above 0.9 for the duration of the fermentation process. Final endpoint pH 5.2 was reached in ~12–17 h, while endpoint pH 4.6 was reached in 20–22 h using the conditions described. Inactivation of *T. gondii* was accomplished well before fermentation to the endpoint pH was reached. At the time of complete inactivation, pH in sampled chubs had reached pH 6.31 in the endpoint pH 4.6 batter, and pH 6.53 in the endpoint pH 5.2 batter.

A summary of the results for complete *T. gondii* inactivation in dry cured sausage indicates the following proposed rule is applicable; the fermentation time has been lowered based upon the current results and those reported in Hill et al. (2018):

If pH is >4.6 and ≤5.2, and % NaCl ≥1.3%, 4 h of fermentation is required to achieve complete inactivation of *T. gondii* bradyzoites (in tissue cysts). This proposed rule is not valid for pH outside of the range (4.6, 5.2), nor for NaCl <1.3%.

## 4 Discussion

Ingestion of meat, especially pork, has been suggested as a major route of transmission of *T. gondii* in the U.S. (Dubey, 2010; Roghmann et al., 1999; Lopez et al., 2000). However, no regulations exist to inactivate *T. gondii* in meats destined for commerce. It has been widely believed, with little direct evidence, that regulated inactivation protocols in place to inactivate *Trichinella spiralis* were sufficient to inactivate *T. gondii* in ready to eat meat products containing pork such as dry cured sausages. Our previous studies demonstrated that the most common dry cure processes approved for inactivation of *T. spiralis* do result in inactivation of *T. gondii* (Hill et al., 2018, Hill et al., 2017), even in the lowest salt concentration tested (1.3%). This is important, since salt concentrations lower than 1.3% are not typically used for preparing dry cured sausage; reduction of water in meat induced by salt (and sugar) reduces microbial activity through dehydration, and extraction of muscle myofibril proteins by these components promotes adhesion of the comminuted meat particles during formulation (Getty, 2005). Salt in excess of 1.3% is typically used for these purposes. It is therefore unlikely that preparations of dry cured sausage would be prepared using formulations that would not inactivate *T. gondii*. The rapidity of inactivation in the pH 4.6 endpoint batter in comparison to the pH 5.2 endpoint batter may be due to the contribution of sugar to more rapid dehydration and inactivation of the pathogen. The pH 4.6 endpoint batter contained 0.7% sugar versus 0.25% sugar in the pH 5.2 endpoint batter, likely contributing to additional reverse osmotic pressure on the bradyzoites in the meat. Neither αw or pH likely significantly contributed to inactivation of *T. gondii* in either batter preparation, since the αw and pH did not change significantly from T-0 until the time of complete inactivation was demonstrated after 2 h.

In a previous report (Hill et al., 2018), 2 mice were found to be serologically and tissue cyst positive for *T. gondii* in 1 of the batter formulations tested after 3 h of fermentation (1.3% NaCl, pH 4.6 endpoint), while in this study, no mice were found infected after the initiation of fermentation in either of the batter formulations tested. However, as in the current study, all subsequent tests for *T. gondii* tissue cysts in brain squashes and ELISA in the earlier study were negative in all batter formulations tested. While these results differ slightly in timing of inactivation, both studies demonstrate the rapidity of inactivation of *T. gondii* bradyzoites in a low salt concentration which is lower than typically used in pork curing processes, and during a stage of production (early fermentation) which would not be accessed by consumers. Results of both studies confirm the safety of ready to eat products containing pork with respect to *T. gondii* prepared using typical NaCl concentrations at or above 1.3%, and industry standard fermentation and drying procedures.

These data provide a practical assessment of meat chemistry parameters which can be used to predict inactivation of *T. gondii* during formulation and curing, and can be used by producers to evaluate current dry cure processes to mitigate the risk of their products to consumers.

In this study, we have described and updated parameters for inactivation of *T. gondii* using a model curing process; these curing parameters fall within a range of processes which are commonly used by the pork industry for a variety of different dry cured RTE products, and can be easily adapted to modify products to changing consumer expectations and expand product choices, especially those involving lower salt options (Guardia et al., 2006).
